# Impact of Delayed Centrifugation on Interleukin 6 Determination in Human Blood

**DOI:** 10.3390/diagnostics15101187

**Published:** 2025-05-08

**Authors:** Hannah L. Sauerwein, Derik F. Hermsen, Detlef Kindgen-Milles, Erik Michael, Johannes C. Fischer, Fritz Boege

**Affiliations:** 1 Central Institute for Clinical Chemistry and Laboratory Diagnostics, Medical Faculty, Heinrich Heine University, University Hospital, Moorenstraße 5, 40225 Düsseldorf, Germany; 2 Clinic for Anesthesiology, Medical Faculty, Heinrich Heine University, University Hospital, Moorenstraße 5, 40225 Düsseldorf, Germany; 3 Institute for Transplantation Diagnostics and Cell Therapeutics, Medical Faculty, Heinrich Heine University, University Hospital, Moorenstraße 5, 40225 Düsseldorf, Germany

**Keywords:** interleukin-6 (IL-6), blood specimen, pre-analytic interference, pre-centrifugation delay, sepsis (septicemia), lipopolysaccharides (LPSs)

## Abstract

**Background/Objectives**: Clinical experience indicates that the determination of interleukin 6 (IL-6) in human blood can vary depending on time span between sample collection and centrifugation. Here, we evaluated confounding effects in various blood specimens. **Methods**: The blood of healthy individuals and critically ill patients was collected in EDTA-, heparin-, and serum collection tubes. Tubes were facultatively incubated (20 °C, 24–48 h) before centrifugation, and IL-6 was measured in the supernatant. **Results**: The preincubation of the blood collection tubes increased the IL-6 values in heparin plasma (in 17/20 samples up to 50-fold) and serum (in 17/20 samples up to 12-fold). These changes were relevant since the normal values were thereby lifted above the upper confidence limit in 12/20 heparin plasma samples and 4/20 serum samples. These IL-6 increases were probably due to in vitro synthesis as opposed to the release of preformed IL-6 from blood cells because subjecting uncentrifuged collection tubes to mechanical cell lyses had negligible effects on IL-6, while incubation with microbial stimulators dramatically increased these values. In the case of EDTA blood, collection tube preincubation induced IL-6 decreases in 17/20 samples from healthy individuals and 20/23 samples from critically ill patients. **Conclusions**: IL-6 determination in heparin plasma and serum is compromised by delayed centrifugation. This effect is relevant for normal values. It increased the number of false high results by >50%. The delayed centrifugation of EDTA blood decreased the IL-6 values, which caused a single false-negative result in 1/43 healthy and critically ill people. The false-negative rate is possibly higher in EDTA blood from non-critically ill out-patients, exhibiting moderately increased IL-6 levels.

## 1. Introduction

Increases in circulating interleukin-6 (IL-6) and its soluble receptor indicate the transition from acute to chronic inflammation [1]. The determination of IL-6 in human plasma or serum has been a long-standing tool in the diagnosis of chronic inflammatory diseases such as rheumatoid arthritis [1]. By now, it also plays a role in the clinical management of various other etiologies, including cancer [2], cardiovascular disease and atherosclerosis [3], COVID-19 progression [4,5], and post-acute COVID-19 vaccination syndrome [6]. In these and other inflammatory diseases, the sensitivity, specificity, and diagnostic value of IL-6 are considered superior to more conventional inflammation markers, such as C-reactive protein or other acute-phase proteins. The growing demand for IL-6 determination in routine health care has prompted the development of new assays suitable for point-of-care testing and high-throughput measurements [7,8]. Despite the widespread implementation of IL-6 measurements in medical laboratories, pre-analytic interferences and disturbances of IL-6 measurements are poorly defined.

The few available publications on IL-6 pre-analytics provide rather ambiguous information. It has been reported that IL-6 is stable in human serum at ambient temperatures for up to 11 days [9]. While reassuring, this observation does not address interferences acting between the acquisition of a blood sample and the separation of the liquid phase from cellular components. This time period, commonly referred to as pre-centrifugation delay, is the least well-controlled segment of the analytical cycle. Published observations indicate that prolonged pre-centrifugation delay, encompassing the exposure of the uncentrifuged blood sample to ambient temperatures for several hours, will alter the results of IL-6 determination significantly. However, it is unclear in what manner the results are thereby affected. On the one hand, it has been reported that IL-6 values were lowered when serum collection tubes were kept at 20 °C for three hours before centrifugation [10]. On the other hand, it has been reported that IL-6 levels were increased in serum upon preincubation (25 °C, 4 h) of the collection tubes before centrifugation [11]. Interestingly, the latter effect was observed with serum collection tubes and, to a lesser extent, with ethylenediamine tetraacetic acid (EDTA) blood collection tubes [11].

To sum up, prolonged pre-centrifugation delay could be a significant confounder of IL-6 measurements, possibly leading to false high or false low values. It remains unclear which of these opposing effects prevails. It could be that not all blood specimens are as susceptible to interference by pre-centrifugation delay with IL-6 determination. However, heparin blood, the type of specimen most commonly used in Germany for IL-6 determination in hospitalized patients, has not been investigated in this respect. Here, we validate the impact of pre-centrifugation delay on IL-6 determination in the types of blood specimen most commonly used in routine laboratory diagnostic, i.e., heparin plasma, EDTA plasma, and serum. This investigation seems particularly useful for gauging the practicability of IL-6 determination in out-patients. Blood samples of out-patients are often subjected to centrifugation delays of uncertain duration. For this reason, IL-6 determination in blood samples from out-patients is currently considered to hold uncertain validity.

## 2. Materials and Methods

### 2.1. Sample Collection

Leftovers of heparin blood, serum collection tubes, and EDTA blood submitted to the laboratory for routine medical diagnostics were used for all experiments and analyses. Based on case records, patient samples were classified as critically ill with sepsis (*N* = 3 females, *N* = 6 males, mean/median age = 61/58 years) or critically ill without sepsis (*N* = 24 females, *N* = 46 males, mean/median age = 61/65 years). Patients were assumed to be critically ill when subjected to intensive care. These cases included recovery from cardiac (*N* = 36) or abdominal surgery (*N* = 6), end-stage cancer excluding leukemia (*N* = 11), acute infection, and severe autoimmune disease (one each). Sepsis was diagnosed according to the ICD-10 classification supported by repeated positive blood cultures. Leftovers of diagnostic blood samples from healthy blood donors (*N* = 29 females, *N* = 12 males, mean/median age = 39/35.5 years) submitted to the laboratory during blood donation served as controls. All samples were anonymized before inclusion. The clinical trial protocols were approved by the local ethics board of Heinrich-Heine University Düsseldorf (study number 2023-2681). This investigation conforms to the principles outlined in the World’s Medical Association’s Declaration of Helsinki. This study reflects a quality control study as stipulated by the German accreditation agency (DAkks).

### 2.2. Laboratory Procedures

Blood samples were collected by cubital vein puncture using the Vacutainer^®^ system (Becton Dickinson GmbH, Heidelberg, Germany). EDTA blood, heparin blood, and serum collection tubes were subjected to centrifugation (4000× *g*, 10 min) within 30 min, and IL-6 was measured in the supernatant. Uncentrifuged blood samples were facultatively subjected to cell lysis by ultrasound treatment using a Bandelin Sonoplus HD 2070 Ultraschall-Homogenisator (1 2 GmbH & Co. KG, Berlin, Germany) or to preincubation (20 °C, 24–72 h). Preincubation was carried out with and without the addition of lipopolysaccharide (LPS) from the *Escherichia coli* strain O55:B5 or phytohemagglutinin (PHA) from *Phaseolus vulgaris* (both SIGMA-ALDRICH, St. Louis, MO, USA). Stock solutions of LPS and PHA were diluted with phosphate-buffered saline (GIBCO/Fisher Scientific, Schwerte, Germany). IL-6 was measured by the Elecsys IL-6 assay implemented on a Cobas 8000 analyzer (both Roche Diagnostics, Mannheim, Germany). Intertest variation was controlled daily at the cut-off and 100-fold cut-off. This was less than 0.1% of the values at both levels and for all types of blood specimens to which the test was applied. The cut-off value for the test (7 pg/mL) reflects the upper confidence limit in normal healthy individuals, as provided by the manufacturer in the CE certificate of the test. The use of the assay as a routine laboratory diagnostic procedure, including an upper normal confidence limit of 7 pg/mL, was approved by DAkks.

### 2.3. Statistics

Graph Pad Prism 9 (Graph Pad Software, Inc., San Diego, CA, USA, Graph Pad Prism 9 for Apple Macintosh, released 2020) was used for the analysis. Normal distribution was tested with the Shapiro–Wilk test. Non-normally distributed data are presented by median values and interquartile ranges. Differences between groups were analyzed with the Mann–Whitney U test (two-tailed). For all tests, statistical significance was assumed at *p* < 0.01.

## 3. Results

### 3.1. Preincubation of Uncentrifuged Heparin Blood at 20 °C Can Increase IL-6 by Several-Fold

**Figure 1:**
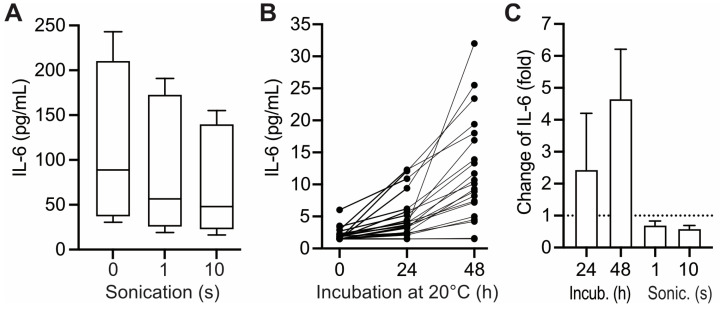
Alteration of plasma IL-6 levels by cell lysis and pre-centrifugation delay. Heparin blood from critically ill patients (*N* = 6) exhibiting increased levels of IL-6 (>7 pg/mL) was facultatively subjected to cell lysis by ultrasound treatment (sonication) for the times indicated ((**A**,**C**) rightmost two columns). Heparin blood from healthy people (*N* = 21) exhibiting normal levels of IL-6 (≤7 pg/mL) was facultatively subjected to incubation at 20 °C for the times indicated ((**B**,C) leftmost two columns). After pre-treatment, samples were centrifuged (4000× *g*, 10 min), and IL-6 was measured in the supernatant. Data in (**A**,**B**): median (line), interquartile range (box), and maximal range (error bars). Columns labeled “0”: results obtained without pre-treatment.

Based on the interleukin-8 paradigm [12], we envisioned that upon prolonged pre-centrifugation delay, IL-6 would possibly increase in the liquid phase of the blood samples due to its release from cell binding in the course of cell lysis. To test this hypothesis, uncentrifuged heparin blood was subjected to mechanical cell lysis via ultrasound treatment. To increase sensitivity, the experiment was carried out with samples from critically ill patients exhibiting increased IL-6 values. Cell lysis was confirmed by an increase in potassium and lactate dehydrogenase and a decrease in haptoglobin. However, cell lysis was not correlated with significant increases in IL-6 in the supernatant. On the contrary, the IL-6 levels dropped slightly upon prolonged cell-disruptive treatment (Figure 1A,C). The release of preformed IL-6 from cells was thus excluded as a mechanism interfering with IL-6 measurements in human blood samples.

Alternatively, we imagined that IL-6 levels could be increased during prolonged pre-centrifugation delay due to synthesis and incretion by the leukocytes present in the sample [13]. To test this hypothesis, heparin blood was incubated at 20 °C before centrifugation, and the IL-6 level was subsequently determined in the supernatant. The test was carried out on heparin blood from healthy controls in order to exclude possible interference with the results by the disease-related pre-conditioning of leukocytes [14,15]. In most samples of heparin blood, the IL-6 values increased upon preincubation of the uncentrifuged blood in a manner correlated to the duration of preincubation (effects up to 48 h are shown in Figure 1B,C). The size of this effect exhibited considerable inter-individual variance: Following a pre-centrifugation delay of 24 h (considered relevant for clinical settings), 19 of the 21 tested samples exhibited significant (1.5- to 4.2-fold) increases in IL-6 (Figure 1C). However, in three of the tested samples, preincubation for more than 24 h was required to obtain relevant effects on subsequent IL-6 measurements, while in two samples, the IL-6 measurement was unaffected by preincubation for up to 48 h (Figure 1B).

### 3.2. IL-6 Synthesis Can Be Stimulated in Uncentrifuged Heparin Blood by Addition of LPS and PHA

IL-6 production can be stimulated in isolated human white blood cells by experimental exposure to pro-inflammatory bacterial toxins such as LPS and PHA [13,16]. We used this approach to test whether the leukocytes present in uncentrifuged heparin blood are similarly capable of responding to experimental exposure to pro-inflammatory stimuli. Upon the addition of PHA or LPS to the uncentrifuged heparin blood, IL-6 increased considerably (Figure S1A). These IL-6 increases were about 1000-fold higher than those observed following preincubation without pro-inflammatory stimuli (compare Figure S1A with Figure 1B; please note that in Figure S1, IL-6 is given in ng/mL). The ED_50_ of LPS in heparin blood (≅2 ng/mL) (Figure S1B, insert) was similar to that previously determined in isolated white blood cells [16]. These observations indicate that leukocytes present in heparin blood exhibit a normal IL-6 response to pro-inflammatory stimuli. Therefore, in vitro synthesis and incretion are a plausible source of the IL-6 increases inducible by the preincubation of heparin blood (Figure 1B).

### 3.3. Critical Illness or Sepsis Is Not Correlated to IL-6 Increases Induced by Preincubation of Heparin Blood

**Figure 2:**
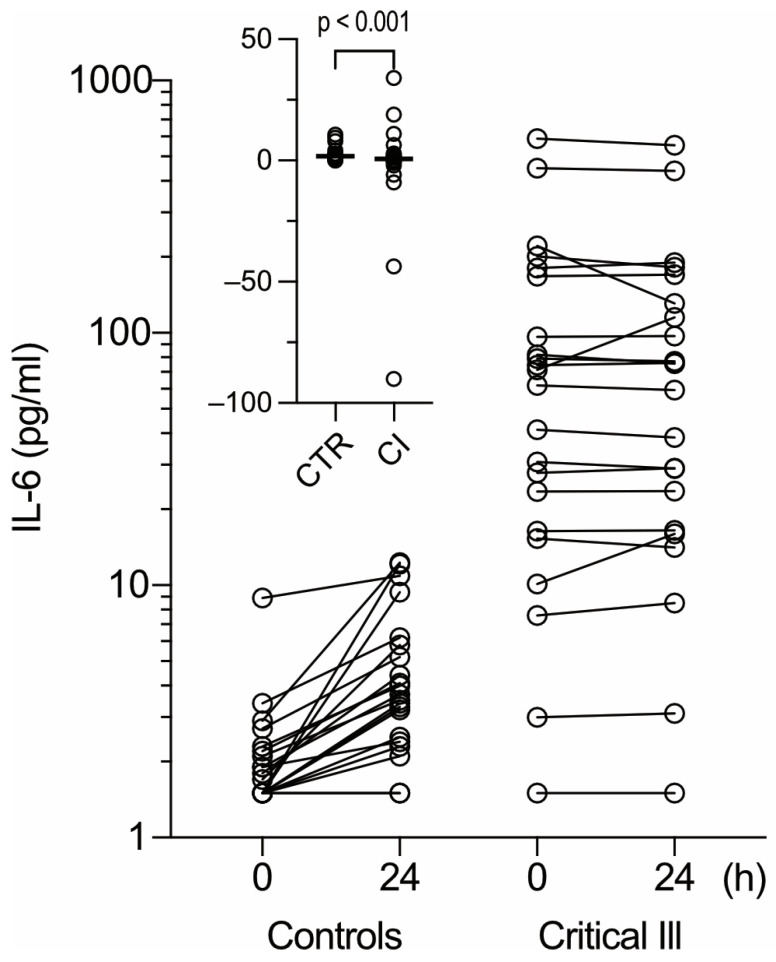
Effect of pre-centrifugation delay on IL-6 in heparin plasma of controls and patients. Heparin blood was facultatively subjected to preincubation at 20 °C. After centrifugation (4000× *g*, 10 min), IL-6 was measured in the supernatant. Values obtained with and without preincubation (24 and 0 h, respectively) for healthy controls (left, *N* = 21) or critically ill patients (right, *N* = 21) are linked by solid lines. Insert: delta of IL-6 values before and after delayed centrifugation obtained in controls (CTR) and critically ill patients (CI). Line: median; bracket: Mann–Whitney U test (two-tailed).

Given the responsiveness of IL-6 production in heparin blood to exogenous LPS, we considered that the pro-inflammatory stimuli present in the blood samples of critically ill patients could possibly be detected via increases in IL-6 following preincubation before centrifugation. However, contrary to expectations, preincubation for 24 of the heparin blood samples from critically ill patients induced lesser increases in, or even decreases in, IL-6 (Figure 2, left). The difference in IL-6’s response to the pre-centrifugation delay between the controls and the critically ill patients was significant in absolute terms (Figure 2, insert) and possibly reflects T-cell dysfunction [14,15]. However, critically ill patients with and without septicemia could not be discriminated between in this way (Figure S2), and a lack of increase in IL-6 upon the preincubation of heparin blood was not correlated with diminished leukocyte counts and/or a lowered lymphocyte fraction.

### 3.4. IL-6 Determination in EDTA Plasma Is Not Affected by Pre-Centrifugation Delay in Clinically Relevant Manner

Similar IL-6 increases as observed here in heparin plasma upon prolonged pre-centrifugation delay (Figure 1) have also recently been observed in serum, whereas IL-6 determination in EDTA plasma seems to be less affected by extended pre-centrifugation delay [11]. Therefore, the type of blood specimen used for the determination of circulating IL-6 could play a role in the validity of the results in cases where centrifugation has been delayed. To follow up on this notion, the impact of pre-centrifugation delay (20 °C, 24 h) on IL-6 determination was compared between the various types of blood specimens commonly employed in German health care (serum, heparin, EDTA blood collection tubes).

**Figure 3:**
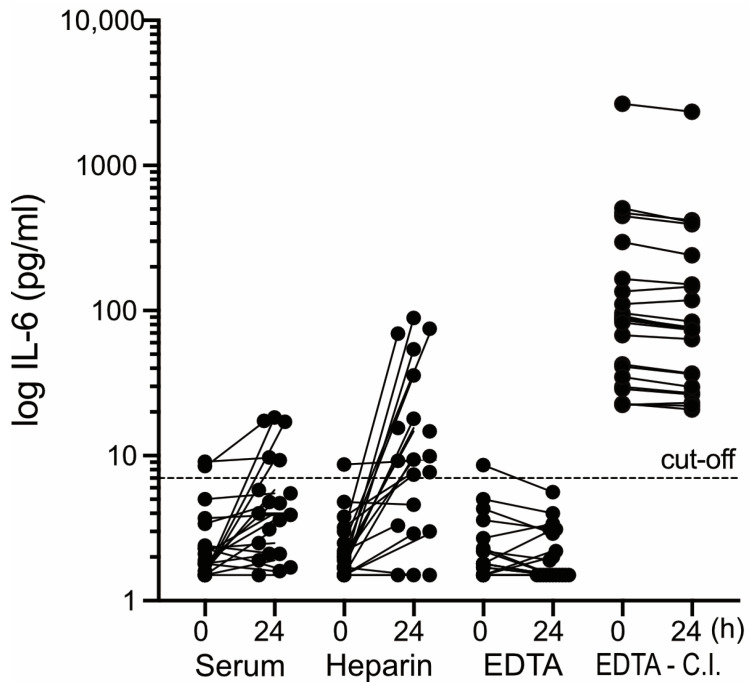
Impact of pre-centrifugation delay on IL-6 determination in different blood specimen. Sets of serum collection tubes, heparin blood (heparin), and EDTA-blood (EDTA) were simultaneously acquired from each of the 24 healthy subjects (6 males, 14 females, aged 29–65, median 43). EDTA blood was also acquired from 23 critically ill patients (23 males, 7 females, aged 46–81, median 66) (EDTA-C.I.). Blood samples were facultatively subjected to preincubation at 20 °C for 24 h. After centrifugation (4000× *g*, 10 min), IL-6 was measured in the supernatant. IL-6 values determined before (0) and after preincubation (24) of a given specimen are linked by solid lines. Dashed line: upper confidence limit.

The comparison was performed on blood samples obtained from presumably healthy controls (10 males, 14 females, aged 29–65, median 43), from whom all four types of blood specimens were gathered with a single vein puncture. Aliquots of the samples were either centrifuged immediately (within 20 min) or after preincubation (20 °C, 24 h). Subsequently, the level of IL-6 was determined in the supernatants. The impact of the simulated centrifugation delay on IL-6 determination differed markedly between the four types of blood specimens (Figure 3). The most pronounced effects were observed in heparin blood (Figure 3, second panel from the left): In 17/20 of the heparin plasma samples tested, IL-6 increased by up to 50-fold following preincubation. Moreover, in 12/20 of the heparin plasma samples, preincubation increased the initially normal IL-6 values above the confidence limit. A similar albeit less pronounced effect was seen in serum (Figure 3, leftmost panel): In 15/20 of the sera tested, the IL-6 values increased by several-fold upon preincubation. However, only in four of these cases were the initially normal values increased above the confidence limit of 7 pg/mL by preincubation. A completely opposite observation was made with EDTA blood (Figure 3, third panel from the left): Preincubation mostly caused a drop in IL-6 values. These alterations were mostly irrelevant from a clinical point of view. In none of the EDTA blood samples obtained from the controls were the normal IL-6 values raised above the confidence limit due to the simulated pre-centrifugation delay. However, in 1/20 of the normal cases, a slightly increased IL-6 value dropped below the confidence limit following the preincubation of EDTA blood, suggesting that extended centrifugation delay can provoke false-negative IL-6 determination in EDTA blood. This effect could be relevant in clinical terms if it induces false-negative IL-6 results in critically ill patients exhibiting IL-6 levels far above the cut-off. To address this issue, the effect of a simulated pre-centrifugation delay on IL-6 was tested in EDTA blood from 23 critically ill patients. As shown in the rightmost panel of Figure 3, the simulated pre-centrifugation delay caused, in 20/23 cases, a decrease in IL-6 of up to 20%. However, in none of these cases was a false-negative result induced by the simulated pre-centrifugation delay.

## 4. Discussion

### 4.1. Salient Findings

- Leukocytes present in heparin blood retain considerable capability to produce IL-6 in response to microbial stimulators;
- Several-fold increases in IL-6 in heparin plasma after prolonged centrifugation delay are most likely caused by ongoing in vitro synthesis;
- IL-6 increases due to centrifugation delay are less frequent/pronounced in the heparin blood of critically ill patients;
- IL-6 increases due to centrifugation delay are less frequent/pronounced in serum collection tubes;
- The preincubation of EDTA blood at 20 °C for 24 h can lead to a drop in IL-6.

### 4.2. Limitations

- The number of patients and controls studied was comparatively small; however, the observed effects were sufficiently large to allow for unambiguous conclusions.
- IL-6 increases following extended centrifugation are assumed to be caused by ongoing in vitro synthesis. This assumption is not supported by direct evidence such as pulse–chase experiments, but it seems to be the most plausible explanation for the observed phenomena.

### 4.3. Remarks

It has been known for some time that the results of IL-6 determination in peripheral blood can vary considerably depending on the time span between sample collection and centrifugation, which prompted the recommendation to “centrifuge tubes quickly following collection” [10] and to adhere to “a standard blood sample handling procedure” [11] when measuring IL-6 in human blood for diagnostic purposes. These recommendations are valuable and worth respecting. However, they are of little help when the pre-centrifugation delay cannot be controlled or avoided, for instance, because a distant laboratory has been commissioned for the measurements. To obtain handling guidelines for the latter situation, we investigated the impact of pre-centrifugation delay on IL-6 values in various specimens of peripheral venous blood.

The results presented here suggest that the risk of obtaining confounding IL-6 values through an extended pre-analytic time span can be avoided or minimized by choosing an appropriate type of blood specimen. In heparin plasma or serum, extended pre-centrifugation delay induced increases in IL-6 in about 85% of cases. These increases were quite substantial (up to 50-fold and 12-fold in heparin plasma and serum, respectively) and caused normal IL-6 values to increase above the confidence limit in 12/20 of the heparin plasma samples and 4/20 of the serum samples. Thus, IL-6 determination in heparin plasma and serum carries a substantial risk of yielding false-positive results if the time-lapse between sample collection and centrifugation is uncertain. Moreover, this risk is higher in healthy subjects than in critically ill patients, which further compromises the disease-discriminative power of the marker.

EDTA blood subjected to extended pre-centrifugation delay seems to carry a much lower risk of false-positive IL-6 results. The preincubation (20 °C, 24 h) of EDTA blood before centrifugation increased the IL-6 values only moderately (less than 2-fold) and infrequently (4/20 samples from controls and 3/23 samples from critically ill patients). In no case were normal IL-6 values thereby pushed above the confidence limit of 7 pg/mL. However, following preincubation, more than half of the EDTA blood samples (12/20) exhibited a notable drop in IL-6 (up to 30%), which in 1/43 of cases pushed a slightly increased IL-6 value below the confidence limit. These observations suggest that extended pre-centrifugation delay possibly enhances the risk of false-negative IL-6 determination in EDTA plasma. False-negative IL-6 results were not induced by extended pre-centrifugation delay in EDTA blood from critically ill patients exhibiting moderately increased IL-6 levels (20–100 pg/mL). However, it cannot be discounted that moderately increased levels of IL-6 in EDTA blood from non-critically ill patients respond to extended centrifugation delay in a different manner, entailing a higher rate of false-negative results.

## 5. Conclusions

When the time span between the collection and centrifugation of blood samples is extended to several hours, IL-6 frequently becomes vastly overestimated in serum or heparin plasma, leading to a high rate of false-positive results. In contrast, IL-6 can be mildly underestimated in EDTA plasma upon delayed centrifugation, which leads to false-negative results in <3% of cases. The lesser confounding effect of delayed centrifugation positions EDTA blood as the specimen of choice for IL-6 determination when the time to centrifugation is unknown or inevitably prolonged. However, it cannot be discounted that moderately increased IL-6 levels are underestimated in EDTA blood from non-critically ill out-patients subjected to extended centrifugation delay.

## Data Availability

The raw data supporting the conclusions of this article will be made available by the authors on request.

## Supplementary Materials

The following supporting information can be downloaded at https://www.mdpi.com/article/10.3390/diagnostics15101187/s1, Figure S1: Impact of LPS and PHA on plasma IL-6. Heparin blood from healthy persons exhibiting normal IL-6 (≤7 pg/mL) was incubated (20 °C, 72 h) with 45 μg/mL PHA (A, left) or with 15 ng/mL LPS (A, right), or with 0.01–15 ng/mL LPS (B) and then centrifuged (4000× *g*, 10 min). IL-6 was measured in the supernatant. Data in A: Corresponding data points (*N* = 10, each) obtained before (CTR) and after pretreatment (PHA, LPS) are linked by solid lines. Data in B: Mean ± SEM, *N* = 5 samples. Insert: Non-linear regression of log-10 transformed data; Figure S2: Effect of pre-centrifugation delay on plasma IL-6 in controls and patients. Heparin blood was facultatively subjected to pre- incubation at 20 °C. After centrifugation (4000× *g*, 10 min), IL-6 was measured in the supernatant. Fold change of IL-6 upon preincubation was derived from the ratio of paired values obtained before and after preincubation. Alteration of IL-6 upon preincubation (72 h). Receiver operator characteristics (dots and solid lines; dotted lines: identity). Left panel: healthy controls (*N* = 15) versus critical ill patients (*N* = 55). Right panel: critical ill patients with (*N* = 9) or without (*N* = 46) sepsis.

## Abbreviations

The following abbreviations are used in this manuscript:

EDTA	Ethylenediamine tetraacetic acid
IL-6	Interleukin 6
LPSs	Lipopolysaccharides
PHA	Phytohemagglutinin
