# Supplementary material for: Impact of Delayed Centrifugation on Interleukin 6 Determination in Human Blood
Source: Diagnostics (Basel). 2025 May 8;15(10):1187. doi: 10.3390/diagnostics15101187 (PMC12109806; doi:10.3390/diagnostics15101187)
Supplement: Supplementary file 1 [file diagnostics-15-01187-s001.zip › diagnostics-3511381-supplementary-update.pdf]

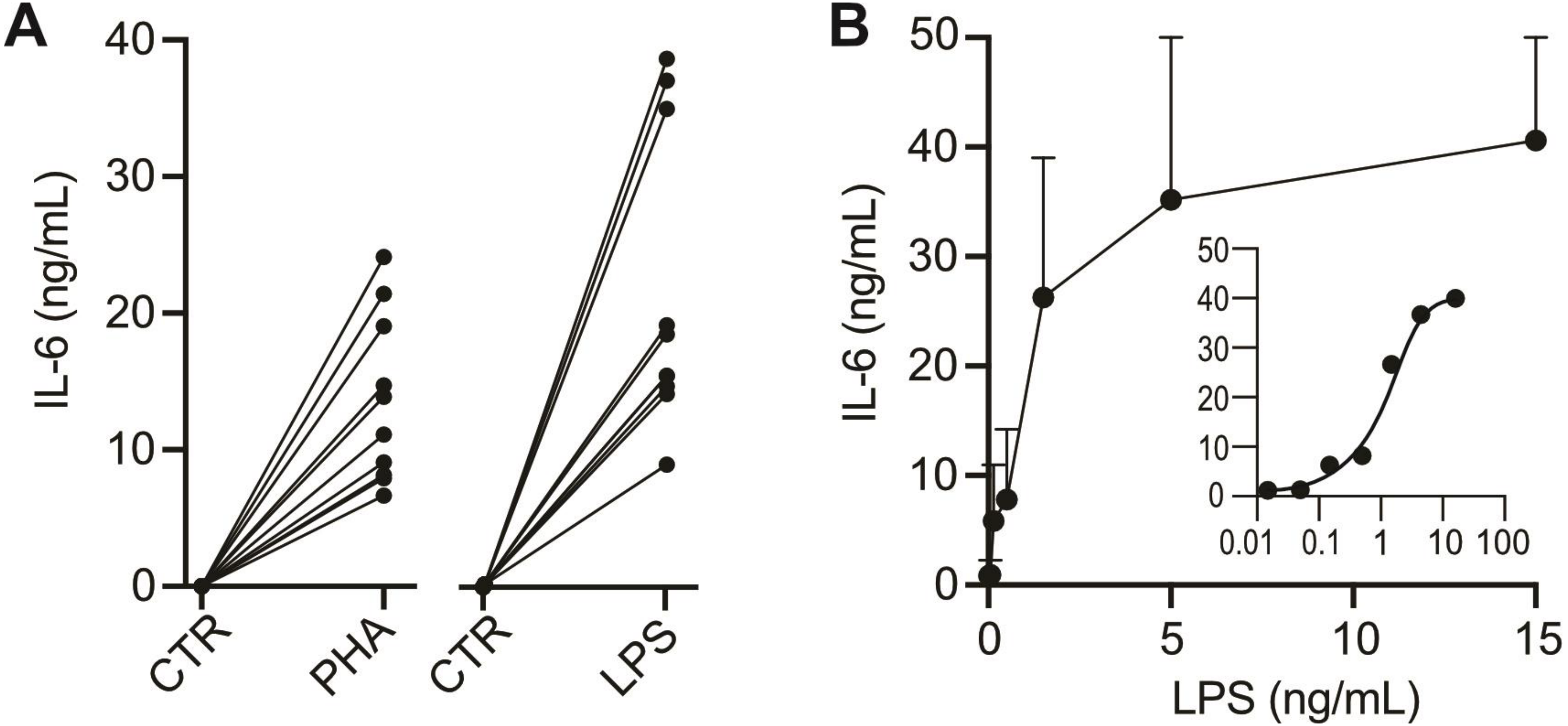

Figure S1. Impact of LPS and PHA on plasma IL-6. Heparin blood from healthy persons exhibiting normal IL-6 (≤ 7pg/mL) was incubated (20 °C, 72 h) with 45 µg/mL PHA (A, left) or with 15 ng/mL LPS (A, right), or with 0.01-15 ng/mL LPS (B) and then centrifuged (4000 x g, 10 min). IL-6 was measured in the supernatant. Data in A: Corresponding data points (N = 10, each) obtained before (CTR) and after pretreatment (PHA, LPS) are linked by solid lines. Data in B: Mean ± SEM, N=5 samples. Insert: Non-linear regression of log-10 transformed data.

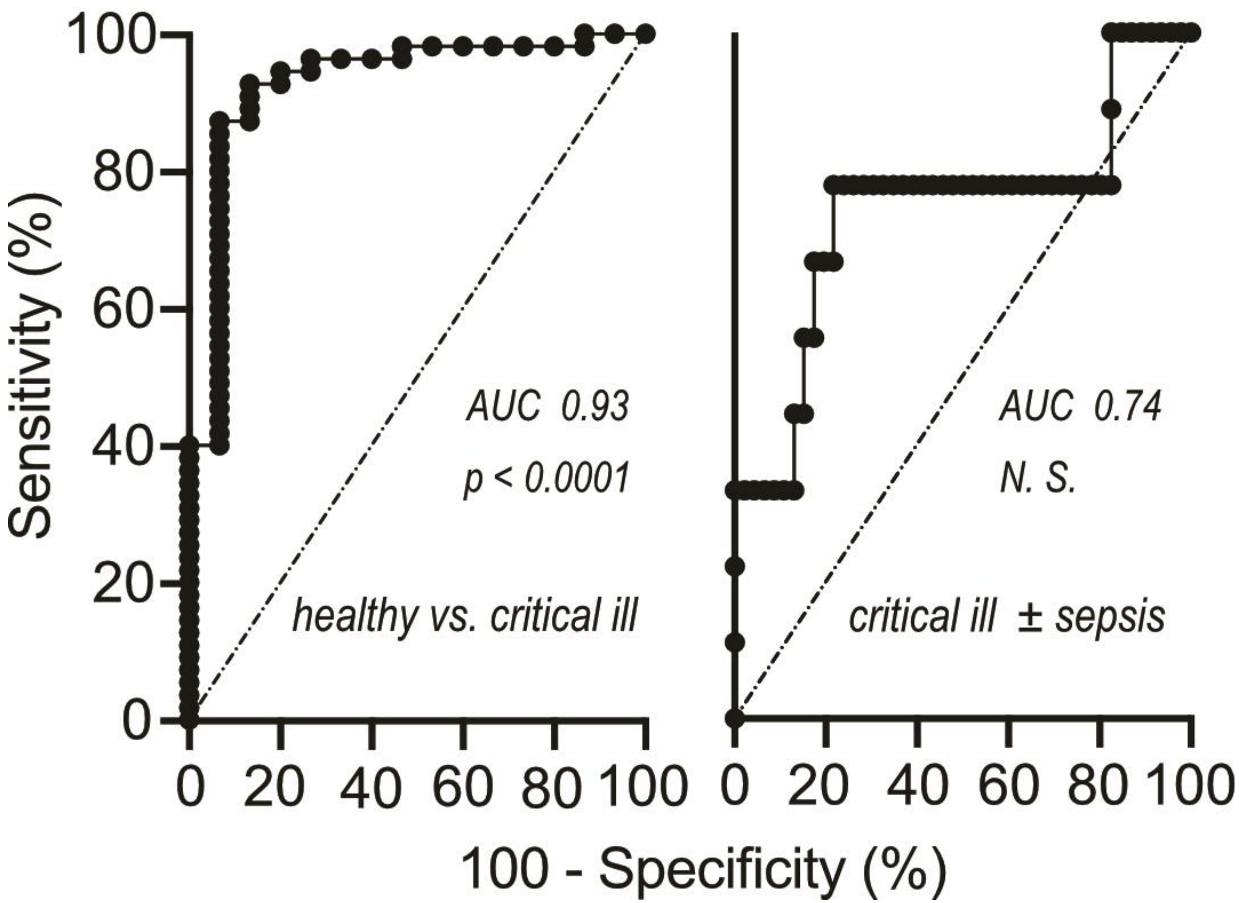

Figure S2. Effect of pre-centrifugation delay on plasma IL-6 in controls and patients. Heparin blood was facultatively subjected to pre- incubation at 20 °C. After centrifugation (4000 x g, 10 min), IL-6 was measured in the supernatant. Fold change of IL-6 upon preincubation was derived from the ratio of paired values obtained before and after preincubation. Alteration of IL-6 upon preincubation (72 h). Receiver operator characteristics (dots and solid lines; dotted lines: identity). Left panel: healthy controls (N = 15) versus critical ill patients (N =55). Right panel: critical ill patients with (N = 9) or without (N=46) sepsis.
